# When and How Will the Epidemic of COVID-19 End?

**DOI:** 10.14336/AD.2021.1120

**Published:** 2022-06-01

**Authors:** Shuo Zhang, Zhen Yang, Zhen-Lin Chen, Zhuo-Ning Li, Shi-Jun Yue, Jia-Jia Li, Fei Yan, Ding-Qiao Xu, Yu-Ping Tang

**Affiliations:** ^1^Key Laboratory of Shaanxi Administration of Traditional Chinese Medicine for TCM Compatibility, and State Key Laboratory of Research & Development of Characteristic Qin Medicine Resources (Cultivation), and Shaanxi Key Laboratory of Chinese Medicine Fundamentals and New Drugs Research, and Shaanxi Collaborative Innovation Center of Chinese Medicinal Resources Industrialization, Shaanxi University of Chinese Medicine, Xi’an 712046, Shaanxi, China.; ^2^School of Clinical Medicine (Guang’anmen Hospital), Beijing University of Chinese Medicine, Beijing 100029, China.; ^3^School of Public Health, Shaanxi University of Chinese Medicine, Xi’an 712046, Shaanxi, China.; ^4^International Programs Office, Shaanxi University of Chinese Medicine, Xi’an 712046, Shaanxi, China

**To the Editor,**

COVID-19 is a severe respiratory disease caused by a viral infection that is highly contagious and deadly [1]. The rapid increase in the number of pneumonia patients has aroused high vigilance from the World Health Organization and has been listed as a public health emergency of international concern [2]. Since its outbreak, COVID-19 has spread rapidly and enveloped most of the world [3]. As of November 18, 2021, COVID-19 has caused 255911487 confirmed cases and 5142787 deaths worldwide (https://voice.baidu.com/act/newpneumonia/newpneumonia/?from=osari_aladin_banner). Moreover, aging will increase the risk of infection, and COVID-19 has a poor prognosis in the elderly, where a large number of deaths occur [4]. In addition to the health effects, the COVID-19 pandemic has caused social, economic and political damage. In order to actively prevent and control the virus mutation and further spread of COVID-19, a large number of therapeutic drugs have emerged in clinic, and various types of COVID-19 vaccines have been gradually developed. However, there is no treatment method that can completely treat or prevent SARS-CoV-2 infection up to now, thus mask wearing, social distancing and isolation still play a major role in the prevention and control of the epidemic [5]. How long will the COVID-19 pandemic last? How will the outbreak end? This is an important issue that people around the world are particularly concerned about.

## The Effect of Drugs on COVID-19

In the early stage of COVID-19 outbreak, early diagnosis, timely reporting, isolation, and supportive treatment are important strategies for combating SARS-CoV-2 infection in the absence of clear and specific treatment plans. Existing drugs are mainly used for symptomatic treatment of COVID-19 patients to prevent further clinical deterioration. The rapid spread of the virus and the rising patient mortality have prompted the global acceleration of interventions to identify potential and available drugs to control and mitigate the outbreak. Based on the replication process of SARS-CoV-2, screening drugs according to targeted viral proteins or host cell receptors, reusing conventional drugs and searching for broad-spectrum antiviral drugs have become the main strategies for developing potential anti-SARS-CoV-2 therapeutics. Therapeutic agents include drugs that target the virus entry process such as ACE2 receptor inhibitors [6], TMPRSS2 inhibitors [7], membrane fusion inhibitor [8] etc.; RNA polymerase inhibitors such as remdesivir [9] and favipiravir [10] etc.; proteinase inhibitors such as lopinavir and ritonavir [11]. Traditional Chinese medicine (TCM) has played a vital role in China’s fight against COVID-19, and treatment is based on the patient’s individuality, as well as seasonal and local conditions. Three Chinese patent medicines and three TCM prescriptions were the key recommended drugs including *Qing-Fei-Pai-Du*, *Hua-Shi-Bai-Du*, *Xuan-Fei-Bai-Du*, *Lian-Hua-Qing-Wen*, *Jin-Hua-Qing-Gan* and *Xue-Bi-Jing*. The unique advantages of TCM multi-target interventions had become an indispensable systemic approach for patients, and significant progress has been made in China’s battle against COVID-19 [12]. However, medication mentioned above can only relieve and improve the clinical symptoms. With the gradual deepening of the understanding of SARS-CoV-2, the continuous research and development of new drugs and the clinical treatment effects are still under further observation. Recently, Molnupiravir (MK-4482, EIDD-2801) has attracted widespread attention as the first oral anti-SARS-CoV-2 agent. Current studies have shown that Molnupiravir is effective in reducing nasopharyngeal SARS-CoV-2 infectious virus and viral RNA with certain safety and tolerability [13], but the specific clinical effect remains to be further observed. Therefore, current drug therapy is mainly symptomatic treatment, which can only relieve the clinical symptoms of COVID-19 patients to a certain extent, decrease the damage of SARS-CoV-2 to the human body, and reduce the probability of the conversion from mild COVID-19 patients to severe. However, there is no specific drug to kill the virus from the source, and even after discharge, there is no guarantee that there will be no recurrence.

## The Effect of Vaccines on COVID-19

As the global epidemic continues to spread, its prevention and control has become a protracted battle, and the alarming spread, and severity of COVID-19 has dominated global attention. Vaccines will play a key role in the ongoing pressure of COVID-19 prevention and control. Depending on the different targets and technologies, various types of COVID-19 vaccines have been developed, including inactivated vaccines, recombinant spike vaccines, viral vector vaccines, RNA vaccines, live attenuated vaccines, virus-like particles vaccines, and so on [14-16]. These vaccines simply differ in their mechanism of action against COVID-19, resulting in different injection procedures, time and doses, but there is no obvious difference in the effectiveness of different types of vaccines against SARS-CoV-2. After injection, they could improve the geometric mean titre and seroconversion rates *in vivo*, and all of them had good immunogenicity and safety, which could significantly improve human immunity to COVID-19 [17-19]. At present, mass vaccination programs have been approved by China, Russia, the United States, Britain, and other countries. However, there are many kinds of vaccines in clinic with different injection doses and procedures, and the tolerance of people in different ages is uncertain. As a result, the exact efficacy and adverse reactions of various vaccines are unclear, and it is difficult to select the optimal vaccine injection program. The vaccination rate and the realization of herd immunity are key issues in the application of vaccines to prevent and control COVID-19. Vaccination has certain effects on reducing the incidence of severe cases of COVID-19, which enables us to better fight against SARS-CoV-2 infections. However, due to the short time to market for COVID-19 vaccine and the lack of long-term observational data, the risk of infection with COVID-19 after vaccination is unclear. At the same time, the adverse reactions after long-term vaccination are still unknown. Some serious vaccine-related adverse reactions may occur long after the vaccine has been put into use, but the time between the discovery of SARS-CoV-2 and the development of COVID-19 vaccine was not long enough. Long-term and extensive studies are needed to determine how long each vaccine lasts after vaccination and whether there will be serious adverse reactions associated with the vaccine. And a common toxic and side effect of vaccine is antibody dependent enhancement (ADE), which is not known up to now in COVID-19 vaccines [20]. As a result, there is a “vaccine hesitancy” in society as many people are reluctant to or delay getting vaccinated against COVID-19 [21]. Older age is an independent risk factor for death and severe infection from COVID-19, and studies have shown that patients over 60 years of age with history of severe diseases are at a higher risk of ARDS and death [22]. As populations age, this group is growing rapidly in all countries, and since the immune response of the elderly is not as good as that of the young, so they need to be prioritized for COVID-19 vaccination. However, most vaccines do not classify the elderly as suitable for vaccination, which may be related to their underlying diseases. And most of the vaccines evaluated in early clinical trials have been administered in healthy individuals, so immunization regimens that work well in healthy people may not be appropriate or adequate for older adults. Moreover, the aging of the immune system of the elderly may limit the protective effectiveness of vaccines against them [23], which brings some difficulties to the protective effect of the vaccine on them. In terms of the development of the global epidemic, the proportion of cases in children has increased significantly. Studies have shown that the SARS-CoV-2 load in the nasopharynx of child patients is equal to or even greater than that of adult patients, in addition, the children population will have more aggregation activities and the infection is more easily spread [24]. Therefore, the children should also be a priority target for vaccination and need greater protection. However, due to the different immune responses of children and adults, there must be many differences from a few months of infants to teenagers, which leads to different doses and times of vaccination and brings some difficulties to vaccine research and development. And the emergence of SARS-CoV-2 mutations could also have a significant impact on the effectiveness of the COVID-19 vaccine. Scientific studies have found that after six months of vaccination, antibodies in the body may be decreased to varying degrees [25]. At this time, booster injections are needed to increase the antibody level up to 5-10 times and greatly improve the immune effect, but they also can not completely prevent infection [26]. In addition, the debate over the fairness of COVID-19 vaccines has been going on for a long time, and the World Health Organization believes that instead of consuming more vaccines, developed countries that already have the lion’s share of vaccines should first ensure global vaccination rates, while a single dose remains hard to obtain in the third world countries [27]. With the gradual decline of antibodies in the body, it may become the norm to get a booster shot for COVID-19, which will perhaps be needed every year in the future. In terms of current situation, global vaccination coverage is uneven, making it difficult to achieve herd immunity; and the effect of continuous vaccination on COVID-19 prevention and control is not entirely predictable.

## SARS-CoV-2 Host Expansion

With the spread of COVID-19, in addition to humans, a growing number of animals have been found to be susceptible to COVID-19 [28]. The continuous increase in the types and numbers of susceptible animals has put more invisible pressure on people to prevent and control the epidemic. A variety of mammals, including cats, dogs, lions, tigers, minks, and ferrets, have been infected through the contact with COVID-19 patients. In addition, rabbits, pigs, foxes, and civet cats have also been identified as possible susceptible hosts of COVID-19 infection in an infection test. Among them, mutations of SARS-CoV-2 have been found in minks and can be transmitted back to humans, leading to further community transmission. In an effort to prevent the spread, a large number of minks have been culled, causing huge economic losses to the industry [29]. In addition to the emergence of SARS-CoV-2 in animals raised by humans, its “wild” host expansion is underway. Wild snow leopards, mountain lions, gorillas and white-tailed deer have also been found to be infected with COVID-19, and rousettus aegyptiacus, callithrix jacchus, macaca fascularis, macaca mulatta, myodes glarolus and peromycus maniculatus may be susceptible to it. Although it is necessary to carry out a large-scale COVID-19 screening for susceptible wildlife to monitor the infections and mutations, the large types and numbers of wildlife make it impossible to screen each animal accurately, which brings great difficulties to the screening work, thus it is difficult to block the spread of COVID-19 among animals. As most terrestrial wild animals have not yet been tested for COVID-19 susceptibility, there is still a lack of research on the susceptibility of marine wildlife (especially marine mammals), and the number of host animals that are susceptible to COVID-19 is unknown. The host extension of SARS-CoV-2 may continue, and frequent human marine activities and wildlife research will increase the risk of human infection. What’s worse, COVID-19 may continue to spread through the ecosystem, resulting in new COVID-19 variants, which poses an unknown threat to human beings.

## How Will the SARS-CoV-2 Die?

COVID-19 is sweeping the world. When and how will it end? Will it evolve into a flu-like human co-existence for a long time? People are very concerned. The outbreak of COVID-19 reminds people of the 1918 influenza pandemic 100 years ago and SARS 17 years ago. The 1918 influenza pandemic was unusually deadly and occurred between January 1918 and December 1920. It affected more than 200 countries, infected about one third of population in the world at that time (about 1.8 billion people), and killed more than 50 million people, making it one of the most fatal epidemics in human history [30]. The end of this pandemic is a complex and tortuous process, as well as the result of a combination of many factors, but it has not ended yet. It is estimated that about a third of the world’s population is infected with the virus, forming an immune barrier. On the other hand, with the extension of the epidemic time and the multi-generational transmission of the virus, its toxicity becomes less virulent or dangerous, and mutates into organisms that can coexist with humans for a long time. In this sense, the pandemic is not over yet, but has achieved coexistence with humans by “compromising and concession”. In 2003, the outbreak of SARS once made people helpless, but the spread of SARS greatly slowed down in the summer that year, and the epidemic basically stopped after summer and autumn. At that time, no effective treatment had been found, possibly because as SARS spread over generations, the virus gradually became less virulent and coexisted with humans, which seemed to “retreat on its own” and never broke out again.

**Table 1:** Information and characteristics of SARS-CoV-2 mutant strains.

WHO label	Pango lineage	GISAID clade/lineage	Nextstrain clade	Earliest documented samples	Date of designation	Characteristic
Alpha	B.1.1.7	GRY (formerly GR/501Y.V1)	201/S:501Y.V1	United Kingdom, Sep-2020	18-Dec-2020	It can more easily bind to cellular receptors, thereby increasing infectivity and infection rates. The rate of transmission is very fast, 50-70% more contagious than the original SARS-CoV-2 and has reached over 50 countries [31].
Beta	B.1.351	GH/501Y.V2	20H/S:501Y.V2	South Africa, May-2020	18-Dec-2020	It has the characteristics of escaping immune system and strong binding with ACE2 receptor, which can reduce the effectiveness of vaccine protection. Even after vaccination, people may be infected, and it also makes some medical treatments difficult. It has spread to 20 countries [32].
Delta	B.1.617.2	G/452R.V3	21A/S:478K	India, Oct-2020	VOI: 4-Apr-2021 VOC: 11-May-2021	The virulence is significantly increased, and it can better bind to ACE2 and has immune escape, resulting in increased infectivity, which was 80% higher than that of the Alpha [33].
Gamma	P.1	GR/501Y.V3	20J/S:501Y.V3	Brazil, Nov-2020	11-Jan-2021	It has a new and stronger ACE binding site which helps the virus escape the immune system, making it more infectious, and it can reduce the protective effectiveness of vaccines and drug treatments, which is likely to cause more harm. And it has a high rate of transmission, as has been found in Japan, Germany and some other countries [34].

The evolution of the epidemic is determined by the nature of virus evolution. With the large-scale outbreak of the epidemic, more toxic strains will disappear as the host humans die, leaving behind less virulent strains, which is also the advantage of virus evolution. Only when the virulence is weakened, it can get more hosts and reproduce for a long time, which is also the common law of most viruses. The COVID-19 pandemic is widespread, rapid and prolonged. Although the outbreak has come and gone, it shows “signs of decline” as time goes by. The amount of virus and transmissible nature of infected patients decreased obviously, and the number of infections and deaths showed a downward trend. Moreover, the peak number of infections and deaths of the outbreak also gradually reduced, and the death rate also decreased and tended to a low and stable state. The daily numbers of COVID-19 cases and deaths, as well as the mortality rates since COVID-19 outbreak are plotted for each country respectively (https://coronavirus.1point3acres.com/zh), which shows the dynamic trend of the epidemic. In addition, there are 11 SARS-CoV-2 mutant strains currently, of which 4 have been reported as variants of concern by WHO (Table 1): the Alpha variant was found in the United Kingdom, the Delta variant in India, the Gamma variant in Brazil and the Beta variant in South Africa [31-34]. And 5 have been reported as variants of interest by WHO: the Epsilon was found in the United States (California), the Eta in the United Kingdom and Nigeria, the Iota in the United States (New York), the Kappa in India and the Zeta in Brazil. However, these virus mutations all took place between May 2020 and November 2020. At present, no new variant strain causing widespread transmission has yet been found, so the mutant ability of SARS-CoV-2 may be gradually diminished, and there will be fewer new variants in the future. It can be seen that the infectivity, virulence and mutation capacity of SARS-CoV-2 may decrease over time and eventually show a lower infectivity and pathogenecity. Even if the COVID-19 is infected, its illness will gradually decrease and may eventually behave like a common cold.

At present, all countries are actively responding to COVID-19. Transmission reduction, drug treatment and vaccination are the three important and effective measures to fight the epidemic. However, drug treatment and vaccination can only control the spread of the virus and reduce the damage to the human body to a certain extent. In addition, the host of SARS-CoV-2 continues to expand, so it is difficult to completely prevent the transmission of the virus among animals. But COVID-19 will also follow the basic laws of virus evolution. With the extension of epidemic time, although SARS-CoV-2 may not necessarily die out, it will gradually become less infectious and virulent over generations of mutations. Even if the mutation occurs, its development will show a slow decay process under the active intervention such as drug treatment and vaccination, and it may eventually mutate into an organism that can coexist with humans for a long time.

According to the current SARS-CoV-2 attenuation process, it is estimated that by the turn of next spring or summer, the infectivity and pathogenicity of the virus will be reduced to the level close to that of the normal influenza, and it is only then that people will be able to resume their way of living and working as they did two years ago. Of course, wearing masks may become the new normal way of life for many people in the future, as studies have found that targeted protection measures during COVID-19 are not only effective in preventing the spread of the epidemic, but also form a deterrent effect on common respiratory diseases [35].
